# Emulsifying dietary fat modulates postprandial endotoxemia associated with chylomicronemia in obese men: a pilot randomized crossover study

**DOI:** 10.1186/s12944-017-0486-6

**Published:** 2017-05-25

**Authors:** Cécile Vors, Jocelyne Drai, Gaëlle Pineau, Martine Laville, Hubert Vidal, Fabienne Laugerette, Marie-Caroline Michalski

**Affiliations:** 1 Univ-Lyon, CarMeN laboratory, INRA U1397, Inserm U1060, Université Claude Bernard Lyon 1, INSA Lyon, Charles Mérieux Medical School, FR-69600 Oullins, France; 2 Centre de Recherche en Nutrition Humaine Rhône-Alpes (CRNH-RA) and Centre Européen pour la Nutrition et la Santé (CENS), Pierre-Bénite, France; 3 Laboratoire de Biochimie, Centre Hospitalier Lyon Sud, Pierre-Bénite, France; 4 The present address of Cécile Vors is: Institut sur la Nutrition et les Aliments Fonctionnels (INAF), Pavillon des Services 2440 boulevard Hochelaga, Québec, G1V 0A6 Canada

## Abstract

**Background:**

Postprandial hyperlipemia is recognized as a major cardio-metabolic risk factor, recently linked to the co-absorption of pro-inflammatory lipopolysaccharides with dietary lipids. This causes endotoxemia that is involved in the pathophysiology of obesity and insulin resistance, but to date the impact of food formulation is unknown. We tested a novel concept that endotoxin absorption can be modulated by fat emulsified structure in the meal, and potentially differently in obese vs. lean men.

**Methods:**

In a randomized controlled crossover study, eight normal-weight and eight obese age-matched healthy men ingested two isocaloric, isolipidic breakfasts of identical composition including 40 g of milk fat that was emulsified or unemulsified. Plasma- and chylomicron-endotoxemia and chylomicron-triglycerides were measured during 8 h after breakfast ingestion.

**Results:**

After emulsion consumption, parallel to an enhanced chylomicronemia, obese subjects presented an early and sharp increase in chylomicron-endotoxemia at 60 min (*P*_time_ = 0.02), which was higher than (i) after spread fat in obese subjects (*P* < 0.05) and (ii) after both spread and emulsified fat in normal-weight subjects (*P* < 0.05). However in obese subjects, the iAUC of plasma endotoxemia over 8 h was lower after emulsion than after spread fat (*P* < 0.05) whereas in NW subjects such reduction of plasma LPS-iAUC was not observed (*P* = 0.67).

**Conclusion:**

This study provides initial evidence that optimizing fat structure in the meal can be part of a dietary strategy to lower the metabolic impact of postprandial endotoxemia in obese men.

**Trial registration:**

Registered at ClinicalTrials.gov #NCT01249378 on July 13, 2010.

## Background

A transient increase of gut-derived circulating pro-inflammatory lipopolysaccharides (LPS), so-called endotoxemia, can occur following consumption of energy-rich meals [1]. Endotoxemia is now recognized as a major contributing factor in obesity-related metabolic disorders associated with inflammation [2]. Its establishment could partly result from repeated daily events of dietary lipid absorption, which allows intestinal absorption of bacterial LPS. The latter are incorporated into chylomicrons in the bloodstream, thereby contributing to postprandial endotoxemia and the onset of low-grade metabolic inflammation [3–5]. We recently demonstrated that postprandial endotoxemia is modulated by ingested fat amount in obese men: they have (i) higher postprandial endotoxemia and (ii) chylomicrons that get more enriched with LPS compared with normal-weight (NW) subjects after a bigger fat load (40 g vs. 10 g) [3]. There is thus a need to explore how fat-meal induced endotoxemia associated with chylomicronemia could be modulated in obese men to limit metabolic disorders.

Importantly, fat can be present in a meal under various physico-chemical structures [6]. We revealed that lipid absorption can be modulated by emulsifying dietary fat, which enhances postprandial lipemia and lipid β-oxidation compared with spread fat [6]. The potential modulation of postprandial endotoxemia by modifying fat structure thus remains to be explored. Moreover, studying fat emulsions becomes a major issue (i) because of the importance of emulsions in everyday and in clinical nutrition and (ii) since recent results point out the negative impact of synthetic emulsifiers on gut microbiota and metabolic syndrome [7].

As part of a large study about fat absorption and postprandial lipemia [6], we therefore performed this pilot study to test the novel hypotheses that emulsifying fat in the meal could modify postprandial endotoxemia at the systemic and chylomicron levels, potentially differently in obese vs. lean men.

## Methods

### Study design

The Lipinflox study was approved by the French Ethics Committee of Lyon Sud-Est-II and AFSSAPS (French National Agency for Medicines and Health Products Safety) and registered at Clinical Trials (#NCT01249378). The primary outcome about the effect of fat structure on postprandial dietary fatty acid handling and metabolism was previously published in a companion paper [6]. Of note, results about the impact of fat amount on postprandial LPS handling were also reported previously [3]. Here we report secondary outcomes of the Lipinflox study on the modulation of postprandial endotoxemia by fat structure. In a crossover randomized controlled design, subjects performed 2 days of metabolic testing separated by at least 3 weeks. After a standardized dinner evening before and an overnight fast, subjects came to the Human Nutrition Research Center Rhône-Alpes (Lyon, France) and consumed one of the 2 test breakfasts containing 40 g of milk fat, 50 g of bread and 160 mL of skimmed milk and providing 251 kcal. Both breakfasts had exactly the same nutritional composition and only differed by fat structure: fat was either spread on the bread or emulsified in the skimmed milk (milk proteins were the natural emulsifiers). Five hours after breakfast ingestion, all subjects consumed the same standardized lunch providing 713 kcal as described previously [6]. Blood samples were collected from an antecubital arm vein through a catheter at baseline and at regular intervals during 8 h after breakfast ingestion. Plasma was separated by centrifugation (1500 g, 10 min, 4 °C) and stored at −80 °C until analysis or at 4 °C for separation of the chylomicron-rich fraction (CMRF). Extreme care was taken to avoid contamination with exogenous LPS during blood and plasma collection [8]. The treatments were randomized according to a random allocation sequence performed by a CRNH-RA biostatistician using Stat® v.11; two randomization lists were generated and stratified over BMI.

### Subjects

**Fig. 1:**
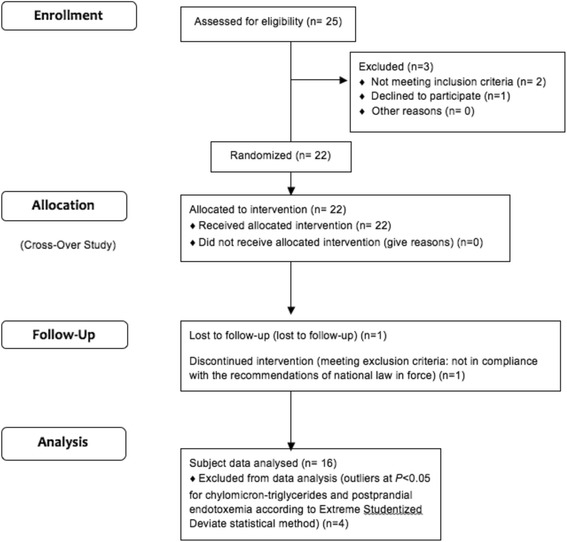
Consort flow diagram of the study participants

The Consort flow diagram of the participants is presented in Fig. 1. On the 20 subjects who completed the study, 16 were finally tested for the secondary outcomes. All subjects were men (8 normal-weight and 8 obese) and gave their written informed consent. All participants were chosen healthy, nonsmokers, free of diabetes and not insulin resistant, not dyslipidemic and without oral inflammatory or infectious phenomena; we specifically excluded people with gingivitis. None was taking any drugs or nutritional supplements affecting lipid metabolism, gut microbiota or inflammation.

### Chylomicron analysis

The chylomicron-rich fractions (CMRF) were collected from plasma by ultracentrifugation (Sorvall Kendro Asheville, USA) as described previously [6]. The procedure was adapted in non-pyrogenic conditions for further endotoxemia analysis [8]. CMRF-TAG concentration was determined using a lipase-glycerokinase method.

### Endotoxemia analysis

Endotoxemia was measured in plasma and CMRF fractions using the LAL assay in kinetic chromogenic conditions (Biogenic, France) [3, 8]. Standard curves presented a correlation coefficient of 0.99 and water was validated as pyrogen-free. To ensure that no inhibition/activation occurred, samples were spiked with 0.05 EU/mL. Spike recovery for plasma and CMRF-samples met standard quality criteria (50–200%).

### Statistical analysis

Data were analyzed using GraphPad Prism software (GraphPad version 5). We calculated that eight subjects per group would provide an 80% power at *P* < 0.05 to detect a 60% difference in endotoxemia according to the emulsified vs. unemulsified structure taking into account a coefficient of variation of 60% according to our previous data in rats [4]. Data normality was checked using the Kolmogorov-Smirnov test. Endotoxemia and chylomicron data followed a normal distribution. The incremental areas under the curve (iAUC; area above baseline fasting value) were calculated by the conventional trapezoid rule from data during the different postprandial periods. Intra-comparisons between test meals were performed using paired Student’s t-test and inter-comparisons between subject groups with unpaired Student’s t-test. Statistical effects of meal (P_meal_), time (P_time_) and their interactions were evaluated on postprandial data of CMRF-LPS using two-way ANOVA followed by Tukey test. Differences were considered significant at the *P* < 0.05 level; *P* < 0.10 were considered as trends due to the limited number of subjects and discussed if necessary.

## Results

Main clinical characteristics of the study participants are presented in Table 1. All subjects were men with comparable mean age, divided into two groups of 8 normal-weight (BMI = 22.3 ± 0.5 kg/m^2^) and 8 obese (BMI = 31.7 ± 0.3 kg/m^2^; waist circumference = 105.6 ± 0.8 cm). Compared to normal-weight (NW), obese volunteers had higher fasting triacylglycerols, CRP and HOMA values as expected but were otherwise healthy (Table 1).

**Table 1:** Fasting clinical characteristics of the study participants

	Normal-weight	Obese	*P* value
*n*	8	8	
Age (y)	29 ± 1	31 ± 2	0.43
Body weight (kg)	72.5 ± 2.1	101.1 ± 2.1	< 0.001
BMI (kg/m^2^)	22.4 ± 0.5	31.8 ± 0.3	< 0.001
Waist circumference (cm)	83.6 ± 1.7	105.6 ± 0.8	< 0.001
HOMA-IR	0.90 ± 0.14	1.75 ± 0.25	0.02
Total cholesterol (mmol/L)	4.90 ± 0.23	5.04 ± 0.21	0.69
HDL cholesterol (mmol/L)	1.53 ± 0.11	1.09 ± 0.06	0.01
LDL cholesterol (mmol/L)	3.06 ± 0.28	3.24 ± 0.18	0.64
Triacylglycerols (mmol/L)	0.86 ± 0.06	1.46 ± 0.18	0.02
LPS (EU/mL)	0.19 ± 0.05	0.18 ± 0.04	0.73
CRP (mg/L)	1.96 ± 0.01	2.98 ± 0.47	0.04
ASAT (UI/L)	26.9 ± 2.5	30.1 ± 2.7	0.44
ALAT (UI/L)	24.9 ± 4.9	43.1 ± 6.2	0.06

Data are mean ± SEM

*ALAT* alanine amino transferase, *ASAT* aspartame aminotransferase, *CRP* C-reactive protein, *HDL* high-density lipoprotein, *HOMA-IR* homeostasic model assessment of insulin resistance, *LDL* low-density lipoprotein

**Fig. 2:**
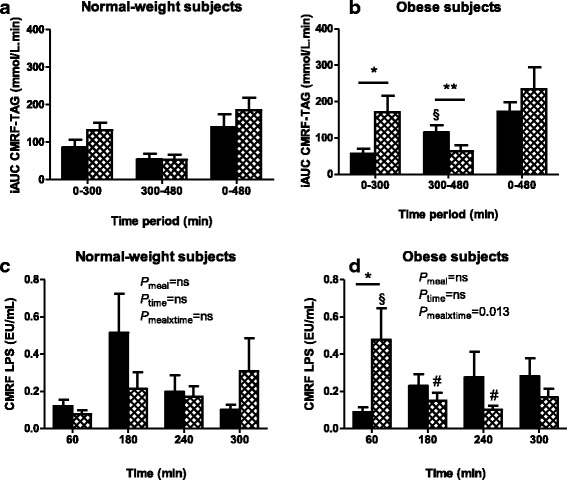
Postprandial chylomicronemia expressed as cumulative iAUC of CMRF-TAG (**a**, **b**) and associated endotoxemia expressed as CMRF-LPS along postprandial time (**c**, **d**) in normal-weight (**a**, **c**) and obese (**b**, **d**) subjects. Black bars identify results after spread fat consumption whereas hatched bars identify results after emulsified fat consumption. **a**-**b** * *P* < 0.05, ** *P* < 0.01, paired Student’s t-test. ^§^ *P* < 0.05, unpaired Student’s t-test vs. NW subjects. **c**-**d** Two-way ANOVA used to test meal and time effects on CMRF-LPS (P_meal_, P_time_ and P_mealxtime_). **d** * *P* < 0.05, paired Student’s t-test. § *P* < 0.05, unpaired Student’s t-test vs. NW subjects. # *P* < 0.10 vs. 60 min, one-way ANOVA followed by Tukey test

In NW subjects, the postprandial iAUC of CMRF-TAG was not significantly affected by fat emulsification (Fig. 2a). In obese subjects, the postprandial iAUC of CMRF-TAG was higher in the 0-300 min period after emulsified fat (*P* < 0.05; Fig. 2b). Postprandial iAUC CMRF-TAG was higher in the 300-480 min period (post-lunch) in obese subjects when spread fat was consumed at breakfast (i) compared to emulsion (*P* < 0.01) and (ii) compared to NW subjects consuming spread fat (*P* < 0.05).

**Fig. 3:**
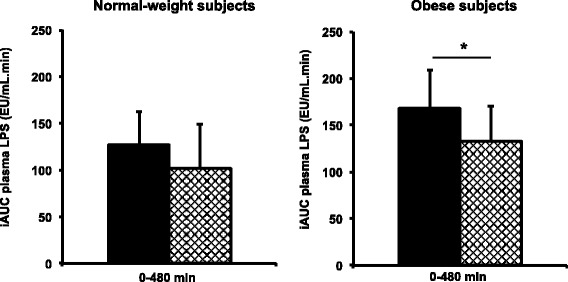
Postprandial cumulative plasma endotoxemia over 8 h in (**a**) normal-weight and **b** obese subjects. *Black bars* identify results after spread fat consumption whereas hatched bars identify results after emulsified fat consumption. **b** * *P* < 0.05 paired Student’s t-test

Regarding CMRF-endotoxemia, no significant meal or time effect was observed in NW subjects (Fig. 2c). In obese subjects, neither meal nor time effect was observed but there was a significant interaction between meal and time (*P* < 0.05, Fig. 2d). After spread fat, CMRF-endotoxemia in obese subjects tended to be higher at 180 min and 300 min vs. 60 min (*P* = 0.06 and *P* = 0.10, respectively; Fig. 2d). After emulsion, obese subjects presented an early and sharp increase in CMRF-endotoxemia at 60 min (P_time_ = 0.02; *P* < 0.05 vs. 180 min and 240 min), which was higher than CMRF-endotoxemia (i) after spread fat in obese subjects (*P* < 0.05) and (ii) after both spread and emulsified fat in NW subjects (*P* < 0.05). In obese subjects, the postprandial plasma accumulation of LPS during the entire exploration day (iAUC 0–480 min) was lower after emulsion than after spread fat (*P* < 0.05, Fig. 3b), while in NW subjects such reduction of plasma LPS-iAUC was not observed (*P* = 0.67, Fig. 3b).

## Discussion

This pilot study provides data suggestive of an impact of fat structure in the meal on the postprandial absorption and handling of LPS in obese men. We show for the first time that emulsified fat consumption enhances an early endotoxin transport by chylomicrons only in obese men, consistent with the stimulation of the early secretion of chylomicrons. Our previous exploration of postprandial lipid metabolism revealed that emulsified fat enhances postprandial lipemia, with a higher and faster appearance of chylomicronemia as soon as 60 min after breakfast [6]. While in obese men the CMRF-endotoxemia was highest and quite constant between 180 and 300 min after spread fat, it appeared as a single peak at 60 min after emulsion. Despite this higher and acute transport of LPS by CMRF after emulsion, there was finally no increase of cumulated plasma endotoxemia over 8 h after emulsion vs. spread fat. We previously reported a better clearance of chylomicrons after emulsion vs. spread fat associated with larger chylomicrons [6]. Larger chylomicrons are more clearable, probably due to a greater affinity for the lipoprotein lipase: such a chylomicron-structure effect could have participated to LPS clearance with the emulsion. Because LPS catabolism could be impaired in type 2 diabetic patients [9], our results suggest that dairy-like emulsion structure could be of great interest in such individuals. In the present study, a natural emulsion in dairy proteins without addition of lipid- or synthetic-type of emulsifiers was tested but comparing the type of emulsifiers is now mandatory [7, 10].

Furthermore, emulsification did not enhance an early endotoxin transport by chylomicrons in NW subjects. The early rise after emulsion in the obese could be due to a higher intraenterocyte and/or intestinal luminal pool of LPS due to a potential upper colonization of the gut with microbiota, as described in morbid obesity [11]. The lipid absorption after emulsion would then provoke an enhanced release of this LPS pool in plasma via the early chylomicron secretion, similar to the early release of exogenous lipids from the previous meal. Moreover, we cannot rule out that the lymphatic flux would be different after meal in obese vs NW subjects, notably because of their low-grade inflammation and of fat emulsification, which could contribute to differential chylomicron and LPS release.

The present results need to be confirmed by long-term clinical trial in a larger population. However, such results form a basis for future clinical studies on the nutritional management of metabolic diseases. The randomized crossover design of this study also makes the present results robust, despite the small number of participants. Furthermore, at this time such results are only valid for men and cannot be extrapolated to women which will deserve further investigation.

## Conclusions

Altogether, these results suggest that emulsifying dietary fat induces a specific dynamic response of postprandial LPS bound to chylomicrons in obese men, which appears more prone to efficient clearance compared with spread fat. This brings promising basis for further research on the metabolic impact of food formulations on target populations at cardiometabolic risk. The clinical perspectives of this study should thus not be underrated. Our new results in the postprandial phase raise the questions of whether (i) daily ingestion of differently formulated fat would result in different long-term chronic inflammatory outcomes, both in the systemic and tissue levels and (ii) the composition and structuring of dietary lipids, including the type and amount of emulsifiers, could be optimized to this aim.

## Abbreviations

AUC: Area under the curve
BMI: Body mass index
CMRF: Chylomicron-rich fraction
iAUC: Incremental AUC
LPS: Lipopolysaccharides
NW: Normal-weight
TAG: Triaclyglycerols
